# Rural Community‐Based Interventions to Improve the Mental Health and Wellbeing of Children and Young People: A Rapid Scoping Review of the Quantitative and Qualitative Evidence

**DOI:** 10.1002/jcop.70037

**Published:** 2025-08-30

**Authors:** Emily McDougal, Ayesha Sheikh, Ediane Santana de Lima, S. Tanya Lereya, Julian Edbrooke‐Childs, Jessica Deighton, Tim Hobbs, Peter Fonagy, Abigail Thompson

**Affiliations:** ^1^ Anna Freud London UK; ^2^ University College London London UK; ^3^ Dartington Service Design Lab Buckfastleigh UK

**Keywords:** community, mental health, review, rural population, youth

## Abstract

This study synthesised evidence on community‐based interventions targeting the mental health and wellbeing of children and young people in rural and remote locations. Scoping review methodology was employed. Searches of six databases were conducted. Titles and abstracts (*N* = 6457) were screened against inclusion and exclusion criteria, followed by full text screening (*N* = 61). Twelve publications reporting 10 unique interventions were identified. Interventions varied in design and delivery, with the majority targeting adolescents and focusing on either prevention (e.g., suicide) or improvement of mental health or wellbeing. Themes identified in the synthesis of intervention outcomes included mood and self‐esteem, resilience and coping, and belonging and social connectedness. Barriers and facilitators to intervention implementation were also identified. Initial evidence suggests positive impacts on youth mental health, wellbeing, and community relationships. However, further research into rural community‐based interventions is needed.

## 1 Introduction

Over the past decade, the mental health of children and young people has emerged as a growing concern (Gunnell et al. 2018; Twenge et al. 2021). Between 2012 and 2018, rates of youth loneliness across 36 countries had nearly doubled, and increased loneliness was negatively correlated with life satisfaction (Twenge et al. 2021), highlighting a need for intervention.

There is a growing evidence base for mental health interventions targeting children and young people. Hudson et al. (2023) reviewed the efficacy of interventions for 4‐to‐9‐year‐olds with emerging mental health needs by synthesising the findings from 55 systematic reviews. They found that targeted interventions led to better outcomes compared with those delivered universally. There was also strong evidence for behavioural and cognitive behavioural interventions in improving general mental health symptoms, including externalising and internalising symptoms.

Evidence for interventions targeting adolescents and young adults has also been synthesised. In an overview of systematic reviews of adolescent mental health interventions, Das et al. (2016) found that group‐based and cognitive behavioural therapy interventions delivered in schools had a positive impact on symptoms of depression and anxiety. They also found that exercise interventions were effective in improving self‐esteem, but that the evidence for their impact on social and emotional wellbeing was mixed. In a review of community‐delivered interventions for 11‐to‐25‐year‐olds, Edbrooke‐Childs et al. (2022) found evidence of positive outcomes on mental health and wellbeing for interventions involving exercise and sports, life skills, social action, creative activities, mentoring and mindfulness.

Although this field of research is growing, the evidence base for mental health interventions often relies on data from urban settings, where the drivers of poor mental health and the range of services differ from rural settings. To improve mental health outcomes for young people in rural areas, it is beneficial to adopt a tailored approach that addresses the unique challenges experienced by this group (Hobbs et al. 2023).

Rural living offers many benefits, such as access to green space and a heightened sense of community (Boyd et al. 2008; Gilbert et al. 2016; Glendinning et al. 2003). Indeed, in a scoping review, Wendelboe‐Nelson et al. (2019) found that almost three‐quarters of relevant studies reported positive associations between green space exposure and mental health or wellbeing for adults. However, young people living in rural or remote locations often face poor mental health outcomes, such as low life satisfaction (Smith and Wesselbaum 2024), high rates of suicide (Fontanella et al. 2015; Probst et al. 2019), and feelings of social isolation (Meek 2008).

Multiple drivers of poor mental health in rural settings have been identified (Fontanella et al. 2015; Centre for Mental Health 2020; Probst et al. 2019). Moreover, rurality is not homogenous, with experiences varying widely depending on specific socioeconomic and environmental circumstances (Afifi et al. 2022). Living in remote locations is often associated with poverty (Bettenhausen et al. 2021; Morales et al. 2020), which is a driver of poor mental health outcomes and an example of the mental health inequalities faced by young people (Alegría et al. 2018). In the UK, pockets of poverty and deprivation often exist near regions of rural wealth (Centre for Mental Health 2020; Pateman 2011). For young people in less affluent settings, proximity to areas of relative wealth can exacerbate feelings of social exclusion (Education Authority 2019; Sadler et al. 2014). This adjacency can obscure the needs of less affluent regions due to a lack of granularity in government tools, ultimately leading to inadequate resourcing (Centre for Mental Health 2020). Missing out on government funding is particularly detrimental as rural regions may be disproportionately impacted by cuts to public spending (Rural England 2019).

Resources are often reduced in rural areas, including facilities for socialising (Education Authority 2019), the quality of educational provision (Drummond 2012), work experience and job opportunities (Cartmel and Furlong 2000; Culliney 2017; Youth Employment UK 2023), and digital connectivity (Centre for Mental Health 2020). Critically, mental health service provision is also reduced in rural regions (Association of Child Psychotherapists 2019; Bettenhausen et al. 2021; Boyd et al. 2007), and where available, it may be difficult for young people to access due to reduced or unreliable transportation (Aisbett et al. 2007). Additionally, the close‐knit nature of rural communities has been linked to a heightened sense of stigma, which may prevent adolescents from seeking help for mental health difficulties (Aisbett et al. 2007; Boyd et al. 2007).

Rural living also disproportionately impacts mental health and wellbeing for marginalised groups of children. The Centre for Mental Health (2020) conducted a review of the impact of rural living in the UK on youth mental health. They identified that those living in poverty, children in care, LGBTQIA+ youth, young carers, children from ethnic minority backgrounds, and disabled children experience additional barriers that mean they are at increased risk of poor mental health. For example, the paucity of safe social spaces in rural areas for LGBTQIA+ youth means that there are very few spaces in which they can be visible and open about their identity.

Some statutory interventions have been adapted to better suit rural locations (e.g., Graham et al. 2021). For example, digital mental health interventions offer a means for clinicians to support patients remotely, minimising the impact of rural barriers and potentially offering a more cost‐effective solution (Mohr et al. 2021). However, non‐statutory interventions also play an important role and may be particularly crucial for rural residents due to the reduced availability of services (Association of Child Psychotherapists 2019; Bettenhausen et al. 2021; Boyd et al. 2007) and increased reliance on non‐statutory supports (Hardy et al. 2024). Non‐statutory and nonclinical approaches often fall under the umbrella term of “community‐based” interventions. The definition of community‐based varies across studies (Castillo et al. 2019; Duncan et al. 2021; Kenny et al. 2014; Pfefferbaum et al. 2013; Public Health England 2015), but may include interventions where community locations such as schools, community‐based organisations or youth provision are the setting for intervention delivery, where members of a community are the target of the intervention (such as family members, peers, local community members), or where the intervention specifically seeks to implement change at the community level (such as strengthening community assets and relationships) (Santana de Lima et al. 2023).

Schools are commonly chosen as a community setting for delivering mental health interventions, in both rural and urban areas. In their systematic review, Clarke et al. (2021) examined the international evidence on the effectiveness of school‐based interventions for adolescent mental health. Their searches returned interventions relating to mental health promotion, prevention of mental health difficulties, and prevention of behavioural difficulties. Effectiveness was variable across interventions, however there was a good evidence base for short‐term impact of social emotional learning interventions on depression and anxiety symptoms, as well as effectiveness of cognitive behavioural therapy interventions on internalising symptoms. However, very few studies included evidence of how interventions might impact young people from underrepresented groups, or for tailoring these interventions for different groups of young people, such as those living rurally. Although a substantial body of literature has addressed community interventions delivered in schools (Clarke et al. 2021), less is known about other types of community‐based interventions, particularly in rural settings. Given the contextual differences for young people living in rural areas, it is vital that the evidence for interventions implemented in rural settings, and their potential effectiveness, is better understood.

For these reasons, this study aimed to conduct a scoping review to collate and summarise rural community‐level interventions implemented to address the mental health needs of children and young people in rural and remote communities over the last 10 years. The review aimed to answer the following research question: What community‐based interventions exist to improve the mental health and wellbeing of children and young people in rural and remote locations? Scoping review methodology was chosen to answer this study question, as the aim was to rapidly identify the types of interventions within this field of interest, as well as identify knowledge gaps (Munn et al. 2018). From the outset it was unclear how many studies would be identified and how variable these would be, however, where information was available, the review also sought to answer the additional sub‐questions: What are the impacts of rural community‐based approaches on the mental health of children and young people? What are the community‐level impacts of rural community‐based approaches to improve mental health? What are the main barriers and facilitators in rural community‐based approaches to improve young people's mental health?

## 2 Methods

### 2.1 Protocol and Registration

The review protocol was pre‐registered on the Open Science Framework (OSF) on 01/03/2024 and can be accessed at the following link: https://osf.io/84a3f.

### 2.2 Eligibility Criteria

Studies were included if they involved children and young people (up to 25 years of age) or discussed the impacts of an intervention on this demographic, and examined at least one community‐based intervention in a rural setting. Definitions of rurality vary, for example, settlements with under 10,000 residents, or areas that are sparsely populated and isolated from facilities or services. For this review, studies were considered to meet the criteria of rurality if they stated “rural” or a synonym in their abstract. A “community‐based” intervention was defined as any non‐statutory intervention (e.g., not part of a government‐funded health service) and not taking place within a school as part of the curriculum or during normal school hours. The definition also included interventions targeting parents, families, and other caregivers, provided that outcomes for children and young people were reported. Studies were included if they reported any of the following: child and young person mental health or wellbeing, community‐level outcomes (e.g., community relationships), or barriers or facilitators of the intervention in improving young people's mental health and wellbeing. Studies were included if they were published in English and reported on primary research, including RCTs, quasi‐experimental designs, qualitative methods, systematic reviews, and meta‐analyses. Both peer‐reviewed and grey literature was eligible for inclusion in the review. Excluded studies were those conducted in lower‐ and middle‐income countries (LMICs), protocols, corrections, addenda, books, and studies published before 2013. Studies with insufficient information for data extraction were also excluded.

### 2.3 Database Searches and Strategy

Searches were conducted in December 2023. Databases searched included: Web of Science (including Medline, Web of Science Core Collection, KCI‐Korean Journal Database, Russian Science Citation Index, ScIELO Citation Index), PsycINFO, Cochrane Library (via Wiley), Epistemonikos, Scopus, and Google Scholar. Studies were identified using search terms related to the key review concepts: children and young people; remote community; intervention type; family, school, and community; emotional, psychological, and behavioural problems; and wellbeing (see Supporting material for the full search string).

### 2.4 Screening Process

**Figure 1:**
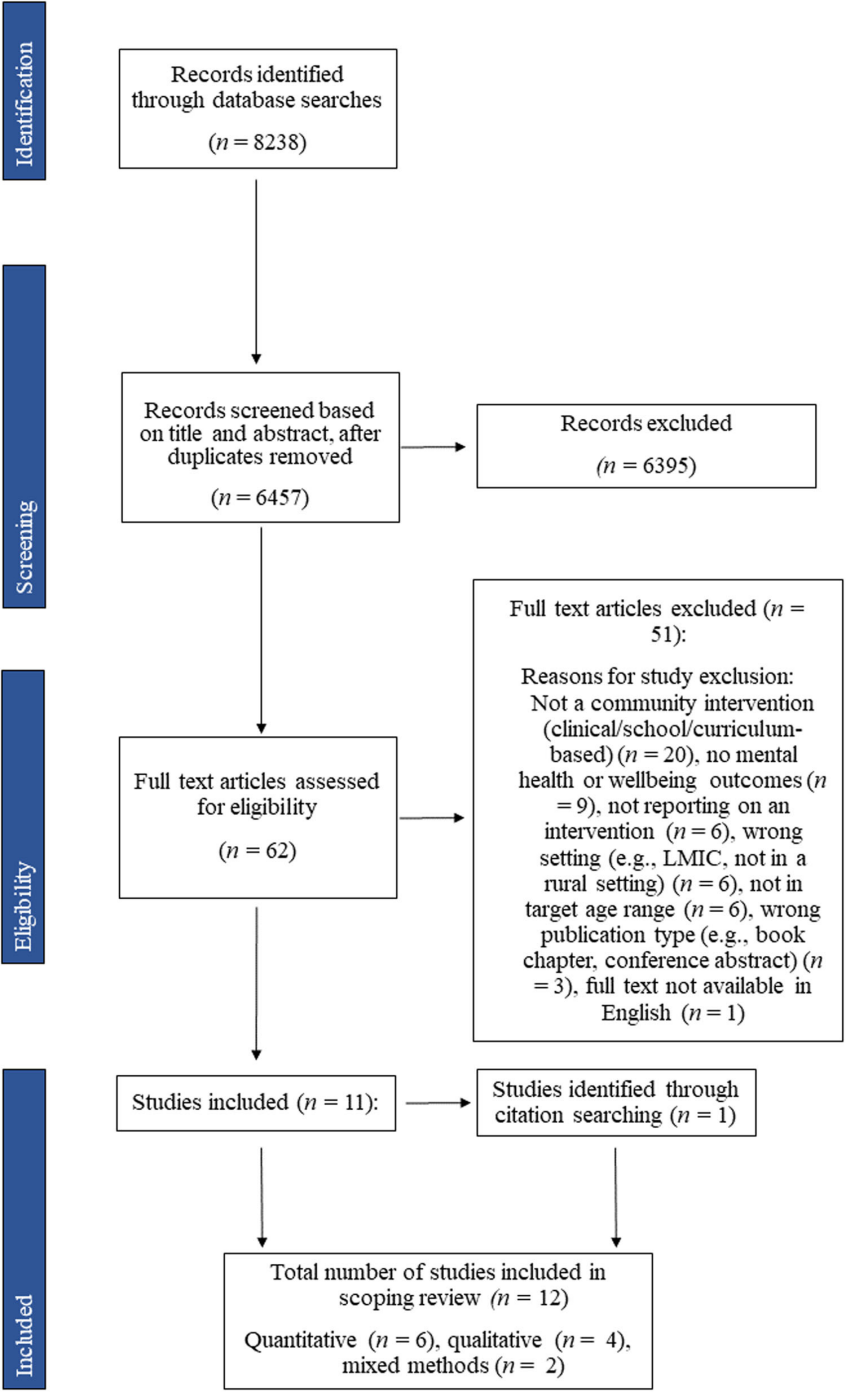
PRISMA flow diagram. Flow diagram illustrating the number of records identified, screened, assessed for eligibility, and included in the scoping review.

The screening process is presented in Figure 1. Database searches resulted in 6457 unique articles to be screened by title and abstract (after removing duplicates). Screening was conducted by three reviewers. The first 25 records were screened by all three reviewers to ensure consensus on inclusion and exclusion procedures. Any conflicts were resolved through discussion with the wider review team. Screening of the remaining papers began when at least 75% agreement on the first 25 records was reached. The remaining records were divided equally among the three reviewers for title and abstract screening. When the abstract was unclear, for example, on whether the study included a community‐based intervention or was delivered as part of a school curriculum, the full paper was retrieved and the intervention description section read for clarification.

From this, 61 articles were provisionally included for full‐text screening. These articles were divided equally between two reviewers, with a third reviewer screening a random sample of 10% of articles to calculate inter‐rater reliability. This fell below 80%, so all full texts were screened by two reviewers. Any conflicts at this stage were resolved through discussion with the wider research team. Full‐text screening resulted in 11 eligible articles. Forwards‐and‐backwards citation searching identified one further eligible article, resulting in a total of 12 articles suitable for inclusion and data extraction.

### 2.5 Data Items for Extraction

Data relevant to the research questions were extracted from the included articles, including variables such as study design, details of the intervention(s), and relevant outcomes. A full list of the variables extracted can be found in the supplementary materials. Data were extracted in this format to allow for a narrative synthesis of results.

## 3 Results

### 3.1 Overview of Included Studies

An overview of the 12 included studies is provided in Table 1. Notably, three cases involved two publications reporting on the same intervention, resulting in a total of 10 interventions represented across the 12 publications. A summary of the intervention characteristics is presented in Table 2.

**Table 1:** Included interventions.

Article No.	Authors, year of publication, country	Intervention name	Intervention aim(s)	Sample size and demographics	Intervention description	Intervention structure	Intervention deliverer(s)	Intervention resources	Reported results of intervention
1	Allen et al. 2018 Alaska, USA	Qungasvik/‘Tools for Life’	Suicide and alcohol misuse prevention, through promotion of protective factors.	*N* = 128 12‐to‐17‐year‐olds 39.8% female, 60.2% male Yup'ik Alaska Natives (100%)	Driven by Indigenous principles. Promoting and teaching protective factors in a Yup'ik culture specific model which is adaptive to local community. Each module of Qungasvik promotes two to four of 13 protective factors.	Delivered in one or more 1–3‐h sessions over several weeks.	Qungasvik researchers alongside local community members and Elders.	Qungasvik intervention manual.	Positive impact on protective factors against suicide. Higher dose intervention was associated with greater growth in these factors, compared to low dose. Provides support for interventions that use cultural approaches.
2	Allen et al. 2023 Alaska, USA			*N* = 239 12‐to‐19‐year‐olds 49% female, 51% male Yup'ik Alaska Natives (100%)					
3	Barnett et al. 2020 Alaska, USA	Culture Camps	Long‐term suicide prevention, through improving youth protective factors and wellness.	*N* = 81 13‐to‐18‐year‐olds 43.25% male, 56.75% female Alaska Natives (100%)	Participation in a variety of group activities, where cultural knowledge and skills are shared. Sessions are also taught on suicide prevention, and life skills (e.g. healthy relationships). Also involves team‐building exercises and time for unsupervised outdoor pursuits.	5‐day long camp.	Elders and group leaders lead cultural/traditional activities. Wellness practitioners teach sessions on suicide/life skills.	Not discussed.	Positive impact on mood, sense of belonging, and resilience.
4	Kogan et al. 2023 Georgia, USA	Strong African American Families (SAAF) programme	Originally for substance abuse prevention. Now to reduce the impact racial discrimination has on the mental health of Black adolescents, by improving effective parenting skills.	*N* = 472 youth, and their caregivers 11‐to‐12‐year‐olds 50.8% female African American or Black (100%)	Family skills training intervention with small groups of caregivers and their adolescents. Structured curriculum targets effective parenting behaviours, adolescent self‐regulation and Black pride.	7 weekly sessions of 2 h (14 h in total). Groups of 4 to 10 families. Delivered in community centres.	Trained Black facilitators, with experience in leading community projects.	SAAF programme manual.	Reduced incidence of depressive symptoms associated with racial discrimination.
5	Brody et al. 2021 Georgia, USA	Strong African American Families‐Teen (SAAF‐T) programme	Family‐centred prevention programme designed to prevent substance use and mental health problems, through skill building for caregivers	*N* = 502, and their caregivers 14‐to‐16‐year‐olds 56.0% girls African American or Black (100%)	Caregivers taught skills such as how to provide emotional and practical support, and how to effectively communicate with youth about sex and alcohol use. Youth learned about goal setting and how resist substance use.	5 consecutive weekly, 2‐h sessions held at community facilities. Youth and caregivers first meet in separate groups, and then meet for joint sessions.	Trained Black facilitators.	Not discussed.	Family skills training weakened the link between racial discrimination and mental health problems.
		Adults in the Making (AIM) programme		*N* = 367, and their caregivers High school seniors 59.1% girls African American or Black (100%)	Training on age‐appropriate caregiving, such as how to provide emotional support, and also problem‐solving and communication skills. Youth were taught strategies to improve their emotional resilience.	6 consecutive weekly, 2‐h sessions. Youth and caregivers first meet in separate groups, and then meet for joint sessions.	Trained Black facilitators.	Not discussed.	Participants who received AIM and experienced racial discrimination showed fewer increases in depressive and anxiety symptoms than control group.
6	Cross and Lauzon 2015 Ontario, Canada	Fusion	To improve rural youth and wellbeing by establishing a youth centre and promoting positive youth development strategies through activities and programmes.	*N* = 18 (9 staff, 3 programme administrators, 6 adult community members) Adults Gender and ethnicity not reported	Wide variety of programmes under one roof, covering art and music, leadership and social development, entrepreneurial and skill development, indoor and outdoor recreational fitness and sports, and a range of technology activities.	Youth are free to participate in whichever activities they choose, whenever they like, which provides autonomy and youth ownership over the centre.	Staff members help to guide activities and provide a source of support for youth.	N/A	Adults reported youth experienced positive impact on sense of self, stress, happiness, and that they were more connected to the local community.
7	Christie and Lauzon 2014 Ontario, Canada			*N* = 12 14‐to‐18‐year‐olds 25% female, 75% male Ethnicity not reported				N/A	Youth reported positive impact on wellbeing and self‐esteem. However, they did not feel valued in wider community.
8	Dowell et al. 2021 Australia	RISE Rugby League Development Programme	Multi‐component youth development programme, aimed at improving youth mental health and wellbeing in young players of organised sport	*N* = 30 (rural sample; *N* = 44 urban sample) 12‐to‐15‐year‐olds 100% male 79.7% white European Australians, 18.9% Australian First Peoples, and 1.4% Maori	Modules delivered to youth in four key areas: grit and optimism; emotional self‐control; social connectedness, and healthy lifestyle habits. Parents/carers and coaches were also sent resources on how to support youth wellbeing.	Face‐to‐face in small groups. Four 30–40‐min sessions, rotated with sporting activities, delivered once a month over four months.	Two co‐facilitators (researchers)	Therapist manual, participant workbook and parent reports and ‘tip sheets’.	Positive impact on anxiety and depression and ability to manage emotions.
9	Jenkins et al. 2018 British Colombia, Canada	Social Networking for Resilience (SONAR)	Mental health promotion through an evidence‐based, youth‐driven programme	*N* = 175 12‐to‐17‐year‐olds 54.9% female, 44.6% male, 0.6% prefer not to say 29.1% Aboriginal or Native, 44.6% White or European, 17.1% mixed race, 6.9% other, and 2.3% missing	Peer researchers created an app to support mental health of their fellow youth. This contained information on youth activities and where they could find safe spaces and further support, and opportunities for youth to share ideas to contribute to local policy etc.	Youth developed the app and organised events to promote its use to their peers and participated in community events to promote broader community engagement and understanding of youth mental health.	Youth peer researchers designed the app. App use was self‐guided by youth users.	SONAR web app.	Negative impact on resilience. Increased participation within community and youth voice more widely valued.
10	Manner et al. 2021 Scotland, UK	Forest School	To improve mental wellbeing by providing a space for adolescent girls to build relationships with each other and improve their emotional intelligence and communication skills, through participation in nature‐based activities.	*N* = 8 12‐to‐13‐year‐olds 100% female Ethnicity not reported	Young people discuss their feelings at the start of each session, and then participate in outdoor activities such as crafts, games, fire‐building and food preparation. The group also discuss how the day went before going back to usual school environment.	Groups of up to 12 young people and four leaders. Weekly sessions of 3 h each, over 12 weeks.	Qualified and experienced Forest School leaders.	Not discussed.	Positive impact on mood and coping with emotions. Positive impact on social skills and community engagement.
11	Umstattd Meyer et al. 2021 Maryland, North Carolina, Oklahoma and Texas, USA	Play Streets	Improve youth mental health and wellbeing and increase community connectedness, by providing a safe space for children to increase their physical activity.	*N* = 46 (14 implementers, 7 adults, 25 children) Grades pre‐K to 6th grade Gender and ethnicity not reported	A street or public space within the community is temporarily closed or repurposed to provide an area for children to play outdoors. Accessing the space is free and young people are able to participate in activities of their choice.	Community leaders and members select the area to be used, for a period of 3‐h, during the summer months.	Community members in rural, low‐income communities.	None, but community organisations received a grant of $6000 to implement programme and buy play materials.	Child participants described how the programme made them feel “happy”. Opportunities were created for enhanced community connection and cohesion.
12	Noel et al. 2013 Florida, USA	TALKnTIME	To prevent the onset of depression in girls, by helping them to gain skills and tools needed to become emotionally healthy, confident, and self‐reliant.	*N* = 12 (assumed–reports number that attended facilitator training but not sample size) USA middle school age girls 100% female	Sessions were guided by cognitive behavioural principles, focusing on reducing negative thoughts and promoting positive youth development ideals such as self‐esteem, resilience, and social skills.	Group intervention, with six participants. 12 weekly sessions lasting 90 min each.	High school peer facilitators recruited from a nearby school. Students were trained and then implemented the intervention in schools.	Not discussed.	Peer facilitators perceived that they had had a positive impact on those who took part in the programme, and that it helped the girls to “feel better about themselves” and “overcome… negative thoughts”.

**Table 2:** Summary table.

Intervention name	Qungasvik^1^^,^ 2	Culture Camps^3^	SAAF, SAAF‐T^4^^,^ 5	AIM^4^	Fusion^6^^,^ 7	Rise^8^	Sonar^9^	Forest school^10^	Play streets^11^	TALKnTIME^12^
**Evaluation design**										
RCT										
Quasi experimental study										
Pilot or feasibility study										
Qualitative case study										
Implementation evaluation										
Participatory research										
**Intervention target population**										
< 5‐year‐olds										
5–10‐year‐olds										
11–18‐year‐olds										
Parents and/or families										
Community members										
**Intervention setting**										
School (extracurricular)										
Community or public space										
Residential trip or retreat										
Club (e.g., sports, youth)										
Online community										
**Intervention delivery**										
Youth/peer facilitation										
Community facilitation										
External facilitators										
Online delivery										
**Intervention content**										
Manualised content										
Unstructured										
Tailored/adaptable										
Group discussions										
Mental health education										
Therapeutic skills										
Functional skills										
Online resources										
Outdoor activities										
Recreational activities										
Cultural activities										
Parenting skills										

*Note:* Blue = evaluation design, Green = intervention target population, Orange = intervention setting, Purple = intervention delivery, Yellow = intervention content.

^1^ Allen et al. (2018).

^2^ Allen et al. (2023).

^3^ Barnett et al. (2020).

^4^ Kogan et al. (2023).

^5^ Brody et al. (2021).

^6^ Cross and Lauzon (2015).

^7^ Christie and Lauzon (2014);

^8^ Dowell et al. (2021).

^9^ Jenkins et al. (2018).

^10^ Manner et al. 2021.

^11^ Umstattd Meyer et al. (2021).

^12^ Noel et al. (2013).

### 3.2 Study Characteristics

Detailed study characteristics are reported in Table 1. The majority of the studies were conducted in the USA (*N* = 7) and Canada (*N* = 3). One study was conducted in Scotland, UK (Manner et al. 2021), and one in Australia (Dowell et al. 2021). All studies reported at least one intervention delivered in a rural setting. Dowell et al. (2021) conducted their study in both urban and rural settings, but reported data separately for each setting, allowing examination of the rural data for this review.

To measure mental health and wellbeing outcomes, six studies used only quantitative methods such as questionnaires, and four used only qualitative methods, including individual interviews and focus groups. Two studies used mixed methods, combining questionnaires and interviews. Quantitative studies varied in their approaches to measuring mental health and/or wellbeing, with some focusing on depressive symptoms and others on anxiety symptoms, general mood, or reasons for living. Most studies using a pre‐post design collected data at two timepoints (baseline and immediate post‐intervention), however two of the studies reported follow up at 18‐34 months post‐baseline (Brody et al. 2021; Kogan et al. 2023).

Study population samples ranged in age from 4 to 19 years, with adolescents being the most frequently reported group. Only one study included participants under 11 years old (Umstattd Meyer et al. 2021). One study with adult participants was included as it reported adult perceptions of youth wellbeing (Cross and Lauzon 2015). Seven studies included participants from more than one gender; other studies either only included single‐gender samples (Dowell et al. 2021; Manner et al. 2021; Noel et al. 2013) or did not report the gender of participants (Cross and Lauzon 2015; Umstattd Meyer et al. 2021). In terms of ethnicity, two studies were conducted with African American and Black participants (Brody et al. 2021; Kogan et al. 2023) and three with Alaska Natives (Allen et al. 2018; Allen et al. 2023; Barnett et al. 2020). Two studies had majority White samples (Dowell et al. 2021; Jenkins et al. 2018). Five studies did not report the ethnicity of their participants.

### 3.3 Definitions of Community‐Based

The classification of interventions as “community‐based” varied. For some, this definition centred around the community setting for intervention delivery, including schools (Noel et al. 2013), rugby clubs (Dowell et al. 2021), outdoor spaces within the neighbourhood (Umstattd Meyer et al. 2021), and community centres (Christie and Lauzon 2014; Cross and Lauzon 2015). Others were classed as ‘community‐based’ because community members were either the target of the intervention (Brody et al. 2021; Kogan et al. 2023) or involved in intervention delivery (Allen et al. 2018; Allen et al. 2023; Barnett et al. 2020). There were no interventions that sought to strengthen community assets; however, some studies aimed to strengthen community relationships (e.g., Allen et al. 2018; Allen et al. 2023; Barnett et al. 2020).

### 3.4 Intervention Aims

A total of 10 unique interventions are represented across the 12 studies, due to three instances where two different papers reported data from the same intervention, and one study that analysed data from two different interventions (Brody et al. 2021). As per the inclusion criteria, youth wellbeing and mental health were reported as outcomes for every intervention; however, the specific aims varied. For example, it was a direct outcome for some interventions but more indirect for others. The characteristics of each intervention are presented in Table 2.

Half of the interventions (*N* = 5) focused on prevention. Three aimed to prevent substance use, including alcohol abuse, and two aimed to prevent youth suicide. The Qungasvik intervention (Allen et al. 2018, 2023) aimed to prevent both alcohol misuse and youth suicide. The TALKnTIME intervention aimed to prevent the onset of major depression (Noel et al. 2013).

The other half of the interventions (*N* = 5) focused on improving or promoting youth mental health and wellbeing. Three of these interventions had additional aims related to community connectedness and civic engagement, and three had additional aims related to skills development.

### 3.5 Intervention Target

All interventions were classed as ‘community‐based’, and in most cases, children and young people were the primary targets. Other interventions targeted families and caregivers, as well as adolescents (Brody et al. 2021; Kogan et al. 2023). Two interventions used participatory models, including youth researchers or facilitators who developed or delivered the intervention (Jenkins et al. 2018; Noel et al. 2013).

### 3.6 Intervention Characteristics

Interventions varied in their delivery format. In Culture Camps (Barnett et al. 2020), young people participated in various group cultural activities, with community elders teaching traditional knowledge and skills. The Qungasvik intervention (Allen et al. 2018; Allen et al. 2023) followed a similar theme, using Indigenous Alaskan approaches and models to inform cultural activities and teach young people protective factors. The Strong African American Families (SAAF) family skills training programme, including the Teen adaptation (SAAF‐T) (Brody et al. 2021; Kogan et al. 2023), incorporated elements of culture, teaching Black pride to adolescents in small groups, alongside effective parenting skills to their caregivers. The Adults in the Making (AIM) programme (Brody et al. 2021) similarly used weekly group sessions to build caregivers' skills in providing emotional support and teaching resilience skills to young people.

Play Streets (Umstattd Meyer et al. 2021) took place within the local neighbourhood, providing young people with safe spaces to play by temporarily closing a street or repurposing an existing space such as a field or car park. For Forest School (Manner et al. 2021), school students were taken to a site away from the school to participate in nature‐based, outdoor pursuits such as crafts, games, and ‘campfire‐style’ activities. Other interventions were based in the school setting but took place after school or outside the usual curriculum. TALKnTIME (Noel et al. 2013) was one example, where peer facilitators delivered a weekly group intervention based on Cognitive Behavioural Therapy (CBT) principles, aiming to promote positive youth development and self‐esteem in adolescent girls. The Social Networking Action for Resilience (SONAR) programme (Jenkins et al. 2018) involved strong youth participation, with peer researchers creating and promoting a mobile app to raise awareness of youth mental health and help them find local activities and forms of support.

Two remaining interventions took place within the wider community and were aimed at larger youth populations. The RISE Rugby League Development intervention (Dowell et al. 2021) took place within youth rugby clubs, utilising a multi‐component approach focusing on different values to improve youth wellbeing, tailored to each group and delivered in monthly sessions. The Fusion Youth and Technology Centre (Fusion) (Christie and Lauzon 2014; Cross and Lauzon 2015) focused on youth development and improving wellbeing through various activities and programmes provided under one roof. Young people who were members of Fusion could choose which activities to participate in, supporting their skills development in a safe and welcoming environment.

### 3.7 Outcomes and Impact

**Figure 2:**
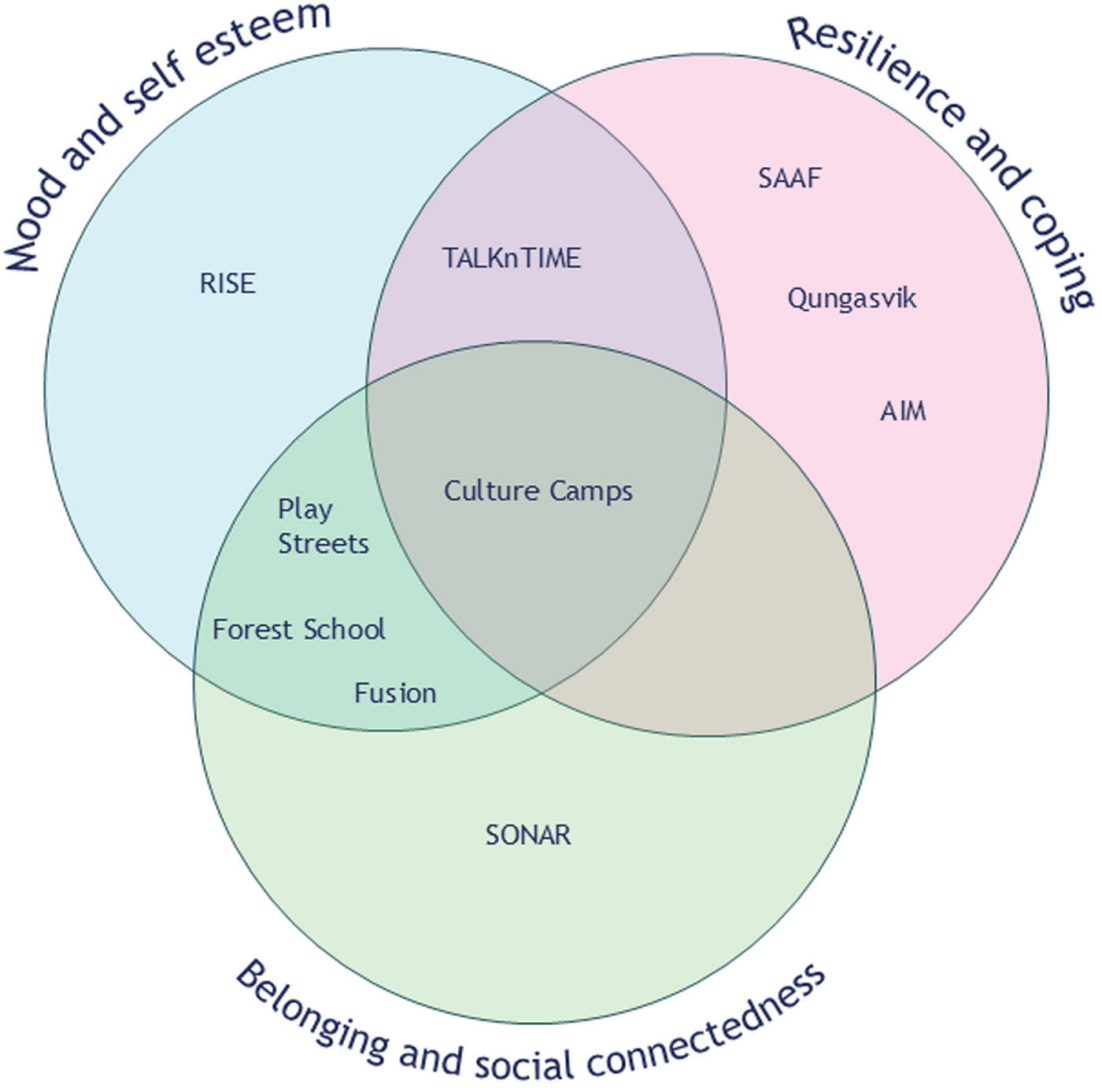
Intervention outcome themes. Venn diagram illustrating how interventions mapped onto three outcome themes: Mood and self‐esteem, resilience and coping, and belonging and social connectedness. Each circle represents one theme, with overlaps indicating interventions that addressed multiple themes. Interventions are abbreviated as follows: SONAR = Social Networking for Resilience; RISE = RISE Rugby League Development Programme; AIM = Adults in the Making; SAAF = Strong African American Families.

Three common themes were identified within the outcomes and impact of the interventions: mood and self‐esteem, resilience and coping, and belonging and social connectedness. Figure 2 shows the distribution of themes across the interventions. Each theme is summarised below. Barriers and facilitators identified within the publications are also discussed.

### 3.8 Mood and Self‐Esteem

Six interventions were associated with positive outcomes related to young people's mood or self‐esteem. In the RISE programme pilot study, symptoms of anxiety and depression were lower after boys participated in the programme (Dowell et al. 2021); anxiety symptoms were significantly reduced (*F*(1,34) = 6.25, *p* = 0.017, *η*2 = 0.16), and reductions in depressive symptoms approached statistical significance (*F*(1,34) = 3.97, *p* = 0.054, *η*2 = 0.11). However, this was the case for boys in both urban and rural settings, with no group differences. In Barnett et al.'s (2020) pilot evaluation of Culture Camps, mood scores increased from pre‐ to post‐assessment with a large effect size (ηp² = 0.24), suggesting that young people experienced more positive feelings after participating in the Culture Camp. However, as there was no control group it is not possible to determine whether this change could be attributed to participation in the intervention. Similarly, in Manner et al.'s (2021) qualitative study of a Forest School programme for girls, participants reported positive changes in their mood, including feeling calmer and happier, as a result of the intervention. Furthermore, in Umstattd Meyer et al. (2021), children reported that Play Streets made them “feel happy”.

Two interventions were reportedly associated with positive outcomes related to self‐esteem, though evidence was limited and lacking in quantitative data. Peer facilitators who delivered TALKnTIME believed that the girls who participated “began to feel better about themselves and think more highly of themselves” (p. 208, Noel et al. 2013). Similarly, the Fusion Youth and Technology Centre created an environment where young people had a positive sense of self; in Christie and Lauzon (2014), young people spoke confidently and positively about themselves in relation to the skills they had developed since visiting Fusion. Adults involved in Fusion also perceived it to have had a positive impact on young people's sense of self (Cross and Lauzon 2015).

### 3.9 Resilience and Coping

Six interventions reported associations between the intervention and young people's resilience and coping. Five of these interventions showed positive associations, but Jenkins et al.'s (2018) assessment of the SONAR intervention reported that resilience worsened from pre‐ to post‐intervention assessment.

In a secondary data analysis of the SAAF programme (Kogan et al. 2023), youth experiences of racial discrimination were associated with statistically significant increases in depressive symptoms in the control group, but not the intervention group. The authors posited that the SAAF programme provides a buffer against the effects of discrimination on mood. However, in Brody et al.'s (2021) evaluation of SAAF‐T, no difference in the impact of racial discrimination on depressive symptoms was found between control and intervention groups, suggesting different versions of the programme may impact depressive symptoms differently.

Brody et al. (2021) also analysed data from the AIM programme, finding that young people who received the intervention and experienced frequent racial discrimination demonstrated less increase in depression and anxiety symptoms compared with those in the control group. There were no group differences in symptoms when experiences of racial discrimination were infrequent, suggesting the AIM programme may provide resilience that buffers against discrimination's negative impact on mental health.

Both evaluations of the Qungasvik intervention found that dosage was associated with changes in “Reasons for Life,” a measure of experiences and beliefs specific to Yup'ik culture that make life meaningful. A higher intensity version of the intervention led to greater improvements, with more Reasons for Life being protective factors against suicide (Allen et al. 2018; Allen et al. 2023). Similarly, the Culture Camp evaluation suggested positive effects on coping with life stressors, as measured by the Self subscale of the Multicultural Mastery Scale, although the effect size was small (ηp² =0.07; Barnett et al. 2020).

In interviews, peer facilitators of TALKnTIME reported that they perceived the intervention to have a positive effect on resilience, with one participant saying the programme “showed them how to be themselves and be strong” (p. 208, Noel et al. 2013).

### 3.10 Belonging and Social Connectedness

Five interventions were reported to positively influence feelings of belonging and/or connectedness within the community. In the pilot evaluation of Culture Camps (Barnett et al. 2020), there was a statistically significant improvement in scores on the Belongingness subscale of the Interpersonal Needs Questionnaire (INQ) from pre‐ to post‐camp, but the effect size was small (ηp² = 0.06). The authors posited that Culture Camps provided an opportunity for young people to develop positive relationships with peers and adults from their community, which increased their sense of belonging and could serve as a protective factor against suicide (Joiner et al. 2009).

In Jenkins et al.'s (2018) evaluation of the SONAR intervention, adult stakeholders reported that relationships between young people and community members changed, with the intervention blurring boundaries between social roles and creating a more connected community.

Interviews with adolescent girls who participated in Forest School (Manner et al. 2021) indicated perceived improvements in social skills and strengthened relationships with family and friends, leading to a stronger desire to spend time with others and increased engagement within their communities. Similarly, Play Streets provided an opportunity for community members to connect (Umstattd Meyer et al. 2021), with participants reflecting that strengthened community bonds facilitated a safe environment for children to play under the shared supervision of the community.

Although there were references to improved feelings of belonging and community connectedness as a result of Fusion, views were mixed. Adults facilitating Fusion perceived positive impacts on community belonging and acceptance for young people (Cross and Lauzon 2015), while young people felt this was true within Fusion but not in the wider community. They felt members of the community did not esteem Fusion highly, leading to continued social exclusion (Christie and Lauzon 2014).

Finally, Allen et al.'s (2018) evaluation of the Qungasvik intervention found no change in young people's perceptions of community support and opportunities (Youth Community Protective Factors Scale).

### 3.11 Barriers and Facilitators

Potential barriers or facilitators were identified for four interventions. In the Forest School intervention (Manner et al. 2021), participants experienced difficulties with group dynamics, such as reluctance to discuss feelings or arguments with other group members. However, arguments reduced over time, and group leaders helped resolve conflicts or used them as learning opportunities. Participants found the outdoor and out‐of‐school elements beneficial, providing more freedom.

The use of peer facilitators in TALKnTIME (Noel et al. 2013) presented potential barriers, such as concerns about time commitments. One participant stated, “I am not sure I will have time next year to commit to it as it requires a lot of time commitment” (p. 208, Noel et al. 2013). In the SONAR intervention (Jenkins et al. 2018), lack of youth engagement was a barrier, as young people did not always use the intervention app. Despite this, the strong element of youth participation was a facilitator, empowering young people by having their voices heard.

Youth empowerment and freedom were also facilitators in Fusion (Christie and Lauzon 2014; Cross and Lauzon 2015), as young people could choose which activities to participate in. Fusion was open to all young people in the community, with fewer financial barriers due to a one‐time payment for lifetime membership. Positive relationships with staff were beneficial, but transitions, such as staff leaving or young people aging out of the service, were challenging. Concerns about community perceptions of Fusion were barriers; if the wider community does not value such interventions, funding and support may wane. Thus, community perceptions need careful consideration for these interventions.

## 4 Discussion

This scoping review aimed to summarise the literature on rural community‐based interventions implemented to address the mental health needs of children and young people in rural and remote communities. It also sought to understand the potential impacts of these interventions on young people's mental health and wellbeing, community‐level impact, and the main barriers and facilitators of rural community‐based approaches to improve young people's mental health. Overall, 12 articles were identified, providing information on 10 different community‐level interventions. The findings are discussed below in relation to the primary and sub‐research questions.

### 4.1 Intervention Characteristics

The 10 interventions identified across twelve studies varied widely in their aims and characteristics, such as intervention length, delivery method, and resources involved. The level of community involvement also varied. Some interventions aimed to enact change at the community level (e.g., Allen et al. 2018; Allen et al. 2023; Barnett et al. 2020), while others targeted young people's parents without seeking broader community change (e.g., Brody et al. 2021; Kogan et al. 2023). The relevance of rurality differed across studies, with some specifically addressing mental health drivers related to rurality (e.g., Christie and Lauzon 2014; Cross and Lauzon 2015), while for others, the rural context was more incidental (e.g., Brody et al. 2021; Jenkins et al. 2018).

### 4.2 Outcomes and Impact

In terms of individual and community‐level outcomes, only one study (Jenkins et al. 2018) reported negative findings. All others reported positive outcomes, grouped into three categories: (1) mood and self‐esteem, (2) resilience and coping, and (3) belonging and social connectedness. Fewer studies reported community‐level outcomes, and for those that did, the outcomes overlapped with individual‐level outcomes in the third category (belonging and social connectedness). These outcomes are promising, especially given that isolation and lack of connectedness have previously been identified as drivers of poor mental health for young people (Allen et al. 2024).

While the reported positive outcomes are promising, caution is warranted in interpreting these findings. As a scoping review, a formal analysis of study quality or publication bias was not conducted. Several included studies were of low methodological quality, with small participant numbers, and only two used a randomised controlled design (to consider attribution of impact). Additionally, only two studies reported long‐term follow‐up, so the persistence of positive benefits over time is largely unknown. This reflects the general lack of evidence‐based research into mental health services and support for young people in rural areas, where services are fewer and gaps in service investments exist (Barclay et al. 2018; Gordon et al. 2023).

### 4.3 Barriers and Facilitators

Only a small number of included articles discussed barriers and facilitators of the interventions for improving young people's mental health (Manner et al. 2021; Noel et al. 2013; Jenkins et al. 2018; Christie and Lauzon 2014; Cross and Lauzon 2015). Some barriers and facilitators were related to intervention features, while others were specific to the community or rural context (or both). Participants in some interventions experienced issues with group dynamics or time commitments, especially peer facilitators. For interventions without structured programmes, poor youth engagement was a barrier. For example, in SONAR, the intervention was implemented via a mobile app in students' own time, a common issue with self‐guided interventions. In a previous study of a school‐based intervention in Norway (Lillevoll et al. 2014), most young people either did not access the online CBT programme or did not proceed past the first few exercises, citing lack of time or forgetting about the programme. Neil et al. (2009) similarly found lower completion rates for an online mental health intervention among adolescents in a community sample compared to their school‐based peers.

If self‐guided community interventions are used to target rural youth, engagement might be improved through closer monitoring by community leaders or other trusted individuals, as suggested for clinical or school‐based interventions (Lillevoll et al. 2014). Remote monitoring, such as phone or video calls, could also benefit parent‐led interventions for rural children (Lyneham and Rapee 2006). In practice, this could involve measures such as community hubs or support groups, where young people who are in receipt of online interventions are able to have less frequent in‐person ‘check‐ins’ in a low‐pressure, familiar environment, to encourage programme adherence. Similar methods could be used to support parents and caregivers who are facilitating interventions for their children, allowing them to receive advice and reassurance when needed.

### 4.4 Supportive Staff as Facilitators and Barriers

Having supportive, relatable staff can act as both a facilitator and a barrier, as highlighted in Fusion (Christie and Lauzon 2014; Cross and Lauzon 2015). Young people were encouraged to attend Fusion due to the valued support from staff members. The importance of building relationships with trusted intervention staff has been noted in previous reviews (Morgan et al. 2020), with positive relationships benefiting the community by supporting social cohesion and capital. However, relationships between staff and young people can become a barrier if youth wellbeing is negatively impacted when staff they trust leave the programme or when young people age out of the service. Additionally, if community perceptions of the programme are poor and young people do not feel valued by the wider community, it can reduce their sense of belonging, which is linked to mental health and wellbeing outcomes (Goswami 2012). This may depend on rural community dynamics, which vary considerably between localities, with some areas having a stronger, more supportive sense of community (Centre for Mental Health 2020), making them more receptive to interventions benefiting all inhabitants. Youth‐ and community‐led coproduction and codesign of mental health interventions may help to address some of these concerns, by allowing interventions to be tailored to suit the needs of each specific community; for example, rural coastal and rural farming communities will face different issues in their localities (Wentworth 2020).

### 4.5 Geographical Gaps in Research

Most of the included studies were based in the USA or Canada, with only one UK‐based and one Australia‐based intervention, highlighting a gap in knowledge regarding effective interventions in European and Australasian rural community contexts. This pattern is common in community‐based, youth‐led research. A review by Branquinho et al. (2020) suggested the need for more research in Europe to understand young people's voices and develop relevant interventions. Rural communities in the UK and Europe differ significantly from those in North America or Australia regarding geographical isolation and access to infrastructure (McAreavey and Brown 2019; Smith et al. 2008). More research is needed to understand how interventions can be adapted or developed for these populations.

Furthermore, this review excluded studies conducted in LMICs due to the differences in context, infrastructure and resources compared with high‐income countries (HICs). This is a significant geographical gap. Indeed, Rose‐Clarke et al. (2024) highlight the lack of youth mental health research in LMICs, despite having the highest mental ill health burden globally. Identifying and understanding the impact of youth mental health interventions in rural communities in LMICs is a vital piece of work, however the research conducted in HICs cannot be extrapolated to this context. Not only are social determinants of mental ill health such as childhood adversity and food insecurity more prevalent for young people living in LMICs, but these are often compounded by increased exposure to natural disasters, political unrest or health epidemics (Rose‐Clarke et al. 2024; Dessauvagie et al. 2020; Vahedi et al. 2024). Generalising research findings from HICs is therefore inappropriate and future research should address this gap.

### 4.6 Ethnicity and Age Group Representation

Several studies did not report the ethnicity of their samples, making it difficult to draw conclusions about the impact of interventions on different ethnic groups or whether further adaptations are needed. Most interventions included adolescents aged 11 to 19 years old, with younger children and older adolescents not represented. This suggests that current community interventions may neglect the needs of younger children and older adolescents, particularly those who have left school. Facilitating interventions for these age groups may be more challenging as young people in rural areas often move away for further study or work (CPRE 2021).

### 4.7 Policy and Practice Implications

Reduced access to resources and opportunities mean rural young people are at risk of being left behind. In 2023, the UK Parliament's Environment, Food and Rural Affairs committee conducted an enquiry into rural mental health and identified an urgent need for national policy to explicitly consider rurality to address this risk (UK Parliament 2023). The findings of the current review complement the enquiry recommendations, highlighting important considerations to be made when designing, resourcing and implementing youth mental health interventions in rural settings.

Given the unique challenges faced by rural youth, strengthening local communities to improve sense of belonging is an important area of focus for policymakers. As reported above, community‐based interventions need to be community‐led and culturally responsive if they are to be relevant and appropriate for those they aim to serve. Furthermore, buy‐in from the community is important for strengthening potential impact. Coproduction or participation methods can be used to ensure that interventions are tailored to the needs of the community. Examples of how to achieve this already exist in the literature. In one example, Santana de Lima et al. (2023) utilised a participatory approach to engaging young people and professionals in defining problems and setting priorities for supporting youth mental health. Engaging communities in priority setting exercises can provide a sense of agency and ownership for young people and community stakeholders (MacLachlan et al. 2024), with the potential to improve engagement. The approach also made space to understand and centre the context of the community taking part, which is vital when developing interventions for rural youth, given the heterogeneity of the population and settings.

Considerations should also be made in relation to delivery models; training and supporting community members to implement the intervention, rather than through external individuals, could lead to whole‐community benefits. This may also contribute to making interventions more culturally relevant, sustainable, and build trust between those delivering and receiving the intervention, all of which are important aspects of mental health interventions. When adopting these delivery models, measures such as community hubs or support groups should be put in place to encourage programme adherence. In the UK, the Government has increased investment in early support hubs to provide better access to wholistic support, including mental health and wellbeing, for young people (Department of Health and Social Care, & Caulfield, M 2024). As demonstrated by the present scoping review, the heterogeneity in audiences, content, and structure of community‐based interventions may continue to pose a challenge about how to evidence their impact and make the case for future investment.

### 4.8 Limitations

Statutory and school‐based interventions were excluded from this review, which may have led to the exclusion of relevant interventions. For example, substantial work targets the mental health of rural adolescents in Australia through community mental health services (Fox et al. 2020; Rickwood et al. 2019), but these were excluded as they were statutory. The concepts of “rurality” and “community” vary widely across the literature (Afifi et al. 2022; Duncan et al. 2021; Castillo et al. 2019; Kenny et al. 2014; Public Health England 2015), and the inclusion criteria depended on the information provided by the authors of each article. Relevant interventions may have been excluded if sufficient information on the rural community setting was not provided.

As this was a scoping review, an inclusive approach was taken in relation to study design criteria, meaning that a variety of different methodologies were included. Although this allowed a wider scoping of the literature, it did limit the ability to conduct a deeper analysis of comparative statistical effectiveness. Additionally, a formal analysis of study quality was not conducted, and an assessment of publication bias cannot be made.

As mentioned above, this review included studies mostly from North America and Canada, highlighting a significant geographical gap and impacting generalisability of the findings to other contexts. It was not feasible to include non‐English language publications in the current review, leading to language bias and a lack of research from a wider range of high‐income countries.

Another clear gap in this review is the lack of evidence around the mental health of marginalised young people in rural settings, such as LGBTQ+ youth, care‐experienced youth, and those in contact with youth justice services. The unique needs and experiences of these groups need to be represented, as tailored interventions can bring more positive mental health outcomes for children and young people (Sheikh et al. 2024). Personalised approaches have previously been found to be underused in minoritised youth (Brannick O Cillin 2022). Future studies should also consider the optimum way to personalise interventions to marginalised groups within a community setting.

## 5 Conclusion

This scoping review sought to summarise interventions aimed at improving the mental health and wellbeing of children and young people in rural and remote settings. Ten interventions across twelve studies were identified and reported. The findings are promising, showing overall positive impacts on mental health and wellbeing and positive community‐level outcomes such as improved community relationships. However, caution is warranted when interpreting these findings, as study quality and publication bias were not formally assessed, and the long‐term impact of these interventions was not reported. Further research is needed to address these gaps and to understand the sustainability of the positive outcomes reported. Policymakers should prioritise capturing the needs of rural communities in relation to mental health, to ensure that young people are not left behind. Participatory approaches offer valuable opportunities to do so equitably.

## Author Contributions

**Emily McDougal:** methodology, investigation, formal analysis, writing – original draft, visualisation. **Ayesha Sheikh:** methodology, investigation, formal analysis, writing – original draft, visualisation. **Ediane Santana de Lima:** conceptualisation, writing – review and editing. **S. Tanya Lereya:** methodology, writing – review and editing. **Julian Edbrooke‐Childs:** conceptualisation, methodology, writing – review and editing. **Jessica Deighton:** conceptualisation, methodology, writing – review and editing. **Tim Hobbs:** conceptualisation, writing – review and editing, supervision. **Peter Fonagy:** writing – review and editing; supervision. **Abigail Thompson:** conceptualisation, investigation, writing – original draft, supervision, project administration.

## Ethics Statement

The authors have nothing to report.

## Conflicts of Interest

The authors declare no conflicts of interest.

## Peer Review

The peer review history for this article is available at https://www.webofscience.com/api/gateway/wos/peer-review/10.1002/jcop.70037.

## Supporting information

Rural review supplementary materials.

## Data Availability

Data sharing not applicable to this article as no datasets were generated or analysed during the current study.
