# Supplementary material for: Rural Community‐Based Interventions to Improve the Mental Health and Wellbeing of Children and Young People: A Rapid Scoping Review of the Quantitative and Qualitative Evidence
Source: J Community Psychol. 2025 Aug 30;53(7):e70037. doi: 10.1002/jcop.70037 (PMC12398394; doi:10.1002/jcop.70037)
Supplement: Supplementary file 1 — Rural review supplementary materials. [file JCOP-53-0-s001.docx]

Example search strategy:

The following search strategy was replicated across all the listed databases: (rural OR outback OR “remote sample” OR “remote population” OR “remote youth” OR “remote children” OR “remote location” OR “remote region” OR “remote setting ” OR “remote families” OR “remote community” OR “remote communities” OR isolated OR countryside OR non?metropolitan OR “underserved area”) AND (“community based” OR “community-based” OR communit* OR parent* OR family OR club OR "support group” OR “youth group” OR church OR religion OR faith OR mosque OR masjid OR temple OR synagogue OR “religious institution” OR club OR centre* OR center OR “civic organi?ation”) AND (program* OR intervention OR “health education” OR campaign OR “social capital” OR “social cohesion” OR “mental health first aid” OR “mental health literacy” OR “stigma” OR “voluntary organisations” OR assets OR “collective action” OR “asset based” OR “asset-based” OR psychoeducation OR class OR classes OR training OR healing OR faith* OR mindfulness OR spiritual* OR meditate* OR retreat OR retreats OR reflexology OR reiki OR massage OR local OR collective OR exercise OR play OR outdoor OR sport* OR walking OR danc* OR yoga OR “tai chi” OR pilates OR “keep fit” OR circuits OR game* OR gaming OR social OR non-clinical OR “non clinical” OR non-medical OR “non medical” OR art OR relaxation OR breathing OR entertainment OR reading OR bibliotherapy OR music OR cinema OR drama OR employment OR volunteering OR hobby OR hobbies OR pets OR creativ* OR drawing OR painting OR garden* OR horticulture OR computer* OR “virtual reality”) AND (“resilience” OR “mental disorder” OR "mental health" OR “mental illness” OR “mental ill health” OR “psychiatric disorder” OR “psychological adjustment” OR “psychological problems” OR “psychopathology” OR wellbeing OR “well being” OR “well-being” OR externali?ing OR internali?ing OR "emotional problem*" OR “emotional adjustment” OR “emotional regulation” OR "emotional difficulties" OR “affective disorder” OR "sense of belonging" OR "wellness" OR "quality of life" OR “self harm” OR self-harm OR “suicide” OR suicidality OR anxiety OR depression OR “depressive symptoms” OR “social isolation” OR loneliness OR “social difficulties” OR “social wellbeing” OR “prosocial behaviour” OR “self-esteem” OR pride OR “positive self-regard" OR “positive youth development” OR mood OR happiness) AND (child* OR adolescen* OR youth OR teenage* OR “young adults” OR “young people” ) From 01/01/2013.

Data extraction variables:

Data was extracted into a table with the following variables: title; author(s); year of publication; type of publication (e.g., research paper, dissertation); research questions/aims; research design (e.g., RCT, feasibility study); source of origin/country; participants (e.g., young people, parents, community members); age range; gender; ethnicity; sample size; intervention name/description; aim of intervention (e.g., suicide prevention); mechanisms of intervention (e.g., direct or indirect, through self-esteem); description of format (e.g., group, 1-2-1); description of content; duration of intervention (i.e., number of sessions, length of sessions, length overall); intervention delivered by (e.g., peers); intervention target group(s) (e.g., youth with depressive symptoms); how are intervention targets selected?; setting intervention is delivered in (e.g., after school club, churches); additional information about intervention; comparator / control group (if applicable); type of data (e.g., quant, qual or both); mental health / wellbeing outcome measures; data collection timepoints; other outcome measures (i.e., incidental); analysis method; what are the impacts of the intervention on CYP mental health?; what are the community-level impacts of the intervention?; what are the main barriers of the intervention (to improve young people's mental health)?; what are the main facilitators of the intervention (to improve young people's mental health)?; implications identified by authors; strengths identified by authors; limitations identified by authors; future directions identified by authors, and conclusions (key messages from the paper).
